# The influences of perfluoroalkyl substances on the rheumatoid arthritis clinic

**DOI:** 10.1186/s12865-022-00483-7

**Published:** 2022-03-04

**Authors:** Yun Zhao, Hangbiao Jin, Jianli Qu, Sunzhao Zhang, Shilei Hu, Jing Xue, Meirong Zhao

**Affiliations:** 1 Department of Rheumatology, Zhejiang University School of Medicine Second Affiliated Hospital, Hangzhou, 310009 Zhejiang People’s Republic of China; 2 Key Laboratory of Microbial Technology for Industrial Pollution Control of Zhejiang Province, College of Environment, Zhejiang University of Technology, Hangzhou, 310032 Zhejiang People’s Republic of China; 3 Department of Laboratory Medicine, Zhejiang University School of Medicine Second Affiliated Hospital, Hangzhou, 310009 Zhejiang People’s Republic of China

**Keywords:** Rheumatoid arthritis, Perfluoroalkyl substances, DAS28, Environmental factor, Visceral lesions

## Abstract

**Background:**

The effect of environmental factors on genetically susceptible individuals is a basic link in the pathogenesis of rheumatoid arthritis. Perfluoroalkyl substances (PFASs) are a class of synthetic organic fluorine chemicals, which have been mass-produced and widely used in the past 60 years, and also have been shown to be one of the major pollutants affecting human health. The impact of fluoride on the development of Rheumatoid Arthritis (RA) is unclear. This study explored the relationship between common fluoride and clinical manifestations of rheumatoid arthritis.

**Results:**

A cohort of 155 patients with RA and 145 health controls in Second Affiliated Hospital of Zhejiang University School of Medicine were investigated. Serum concentrations of all fluoride detected were higher in RA patients than in healthy controls. There were 43 male patients and 112 female patients in the RA cohort. Some of perfluoroalkyl substances (perfluorooctanoate (PFOA), perfluorononanoate (PFNA), perfluorotrdecanoate (PFTrA), perfluorooctanesulfonate (PFOS)) were correlated negatively with the Body Mass Index (BMI); some of them (PFOA, PFNA, PFTrA, PFOS, 8:2 Chlorinated polyfluorinated ether sulfonate (8:2Cl-PFESA)) were correlated positively with the Disease Activity Score 28 (DAS28); two (PFOA, PFOS) of them were correlated positively with the white blood cell count, and one (Perfluoroundecanoate (PFUnA)) of them was correlated negatively with the hemoglobin; two (Perfluorodecanoate (PFDA), PFUnA) of them were correlated negatively with the presence of interstitial lung disease.

**Conclusion:**

These data suggest that exposure to perfluoroalkyl substances may promote the disease activity of rheumatoid arthritis and the visceral lesions.

**Supplementary Information:**

The online version contains supplementary material available at 10.1186/s12865-022-00483-7.

## Background

Rheumatoid arthritis (RA) is one of the classical systemic autoimmune diseases mainly involving joints. The disease affects approximately 0.5–1% of different populations around the world [1]. The pathogenesis of rheumatoid arthritis has not yet been fully elucidated, but the mainstream believes that environmental factors act on individuals with genetic susceptibility to cause immune disorders, leading to the occurrence of joint synovial inflammation and tumor-like proliferation, leading to the destruction of articular cartilage and bone, is the basic mechanism of rheumatoid arthritis. Many genes contribute to increase the susceptibility to rheumatoid arthritis [2], including human leukocyte antigen (HLA) (such as HLA-DRB1) [3] coding genes and non-HLA coding genes (such as Protein tyrosine phosphatase non-receptor type 22 (PTPN22), Peptidylarginine deiminase 4 (PADI4)) [4–6]. The current basic research on rheumatoid arthritis is mainly focused on the search for related genetic factors and the characteristics of immune disorders, while the research on environmental factors is relatively less. Some studies showed that smoking and other ways of bronchial damage (e.g., exposure to silica) increase the risk of rheumatoid arthritis among persons with susceptibility HLA-DR4 alleles [7, 8]. Environmental factors causing damage to pulmonary and other barrier tissues may promote post-translational modifications, through PADI4, that result in citrullination increase of mucosal proteins. Loss of tolerance to such neoepitopes upregulates an anti-citrullinated peptide antibody (ACPA) response beyond the endurance range of the body [9, 10]. Some other studies showed infectious agents (e.g., Epstein-Barr virus, cytomegalovirus, proteus species, and Escherichia coli) and their components (e.g., heat-shock proteins) were associated with rheumatoid arthritis, and molecular mimicry is postulated [11, 12]. Some studies also showed that periodontitis is capable of promoting citrullination of mammalian proteins, thus were associated with RA [13, 14]. Finally, intestinal flora dysbiosis and barrier leakage are closely related to the occurrence of rheumatoid arthritis [15–17].

There is growing concern about the effects of chemical compounds on health. Perfluoroalkyl substances (PFASs), which have been mass-produced and widely used in hundreds of commercial and industrial fields in the past 60 years, are a class of synthetic organic fluorine chemicals with high thermal activity, high chemical stabilities and high surface activity [18]. According to the different polar head group R, PFASs can be divided into different species. Many publications have showed that PFASs could cause a series of health issues. Researchers from the National Health and Nutrition Examination Survey (NHANES) found that most of the participants had measurable PFOS and PFOA concentrations in their bodies [19, 20]. Because of their extremely stable physicochemical properties, PFASs are ubiquitous in the environment, including drinking water, dust, and even in remote Arctic regions [21, 22]; on the other hand, PFASs are virtually non-biodegradable and bind readily to serum proteins in human blood, so they can remain persistently in organisms [23]. Maestri et al. reported that PFAS accumulates more easily in protein-rich areas such as blood and liver [24]. Moreover, some studies on animal toxicology showed that PFOS could induce some specific genes expressing differentially, thus disturb some functional pathways and cause damage to the cytoskeleton organization and the connections of Sertoli cell–cell [25]. Some studies have reported the link between residual PFOA or PFOS and thyroid disease in humans [26, 27]. But the effect of PFASs on autoimmune diseases has not been studied.

In this study, a longitudinal clinical cohort of 155 patients with RA was investigated in Second Affiliated Hospital of Zhejiang University School of Medicine. Serum samples were collected from these participants during the clinic visit and analyzed for PFOA, PFNA, PFDA, PFUnA, PFDoA, PFTrA, PFOS, 8:2Cl-PFESA. The main focus of this study were to explore the association between PFASs in RA patients and the clinic of RA.

## Results

### Demographic, clinical, and immunologic features of 155 patients with rheumatoid arthritis

The patient cohort comprised 155 individuals, including 43 (27.74%) men and 112 (72.26%) women (male: female, ratio, 1:2.6), with a mean age at onset of 45 ± 12 years (range 24–67 year). The mean disease duration was 12 ± 2 years (range, 17–21 year). According to the disease activity assess in rheumatoid arthritis assessed by the number of swelling and tenderness in specific 28 joints, named Disease Activity Score 28 based on CRP (DAS28-CRP), there were 33 (21.3%) patients with disease inactivity, 44 (28.4%) patients with low disease activity, 56 (36.1%) patients with moderate disease activity, and 22 (14.2%) patients with high disease activity. According to the blood routing examination, there were 69 (44.52%) patients with anemia,  12 (7.74%) patients with leucopenia, and no patient with thrombocytopenia. Some patients had mild liver dysfunction and none had kidney dysfunction. There were 143 (92.26%) patients and 118 (76.13%) patients with the disease marker ACPA and Rheumatoid Factor (RF) respectively. The detailed data was shown in the Table 1. Serum concentrations of all fluoride detected were higher in RA patients than in healthy controls (Additional file 1: Table S1).

**Table 1:** Demographic, clinical, and immunologic features of 155 chinese patients with RA

Variables at protocol*	n = 155
Gender, male, n(%)	43 (27.74)
BMI, mean (S.D.), kg/m^2^	22.1 ± 3.4
Age at onset, mean (S.D.), years	45 ± 12
Disease duration, mean (S.D.), years	12 ± 2
DAS28, n(%)	
< 2.6	33(21.3)
2.6 ~ 3.2	44(28.4)
3.2 ~ 5.1	56(36.1)
> 5.1	22(14.2)
Cytopenia	
Anemia (Hb < 110 g/l),n (%)	69(44.52)
Leucopenia (< 4 × 10^9^/L), n (%)	12 (7.74)
Thrombocytopenia (< 100 × 10^9^/L), n (%)	0(0)
Lymphopenia (< 0.8 × 10^9^/L), n (%)	13 (8.39)
High ALT (> 50 U/L), n (%)	0(0)
High AST (> 35 U/L), n (%)	6(3.87)
High TBlL (> 21 umol/L), n (%)	0(0)
High ALP (> 125 U/L), n (%)	14(9.03)
High GGT (> 60 U/L), n (%)	20(12.9)
High Cr (> 104 umol/L), n (%)	0(0)
Interstitial lung disease, n (%)	27(17.42)
Anti-CCP antibody positive, n (%) 143 (92.26)	
RF positive, n (%)	118(76.13)
ILD, n (%)	27(17.4)

*Mean (M) ± standard deviation (S.D) was used to describe the measurement data, and percentage was used to describe the counting data

*RA* rheumatoid arthritis, *HCs* healthy controls, *BMI* body mass index, *DAS28* disease activity score in 28 joints based on c reactive protein, *Hb* hemoglobin, *ALT* alanine transaminase, *AST* aspartate amino transferase, *TBIL* total bilirubin, *GGT* gamma-glutamyl transpeptidase, *Cr* creatinine, *Anti-CCP* anti-cyclic citrullinated peptide, *RF* rheumatoid factor, ILD interstitial lung disease

### Correlation between fluorides and disease activity score 28 of rheumatoid arthritis

The disease activity of patients with RA was scored by DAS28-CRP, and the patients were divided into four groups according to DAS28, disease inactivity group (DAS28 < 2.6), low disease activity group (2.6 ≤ DAS28 < 3.2), moderate disease activity group (3.2 ≤ DAS28 < 5.1) and high disease activity group (5.1 ≤ DAS28) respectively. The median serum concentration of PFOA (3.58 ng/mL, 6.00 ng/mL, 12.74 ng/mL, and 19.78 ng/mL), PFNA (1.08 ng/mL, 1.27 ng/mL, 1.35 ng/mL, 1.72 ng/mL), PFOS (2.43 ng/mL, 3.33 ng/mL, 6.01 ng/mL, 6.81 ng/mL) and 8:2Cl-PFESA (0.18 ng/mL, 0.27 ng/mL, 0.42 ng/mL, 0.57 ng/mL) in disease inactivity group, low disease activity group, moderate disease activity group and high disease activity group reached statistical difference with all p = 0.0001. But the median serum concentration of PFDA (p = 0.0570), PFUnA (p = 0.3750), and PFDoA (p = 0.6496) was not different statistically in different groups. Although it was different statistically (p = 0.0056), the median serum concentration of PFTrA, was 0.14 ng/mL, 0.09 ng/mL, 0.10 ng/mL and 0.12 ng/mL in disease inactivity group, low disease activity group, moderate disease activity group and high disease activity group respectively, did not correlated linearly with the increase of DAS28. The median serum concentration of PFDA, PFUnA and PFDoA did not reach the statistical difference in different DAS28 groups with p = 0.0570, p = 0.3750 and p = 0.6496 respectively. The detailed data was shown in the Table 2.

**Table 2:** Correlation between fluorides and Disease Activity Score 28 of RA

	PFOA (ng/mL)	PFNA (ng/mL)	PFDA (ng/mL)	PFUnA (ng/mL)	PFDoA (ng/mL)	PFTrA (ng/mL)	PFOS (ng/mL)	8:2Cl-PFESA (ng/mL)
DAS28 < 2.6	3.58(2.80 ~ 5.05)	1.08(0.58 ~ 1.45)	0.77(0.47 ~ 1.10)	0.31(0.20 ~ 0.45)	0.09(0.08 ~ 0.14)	0.14(0.11 ~ 0.16)	2.43(1.78 ~ 3.54)	0.18(0.12 ~ 0.49)
2.6 ≤ DAS28 < 3.2	6.00(5.01 ~ 6.66)	1.27(0.71 ~ 2.28)	0.93(0.46 ~ 1.32)	0.36(0.26 ~ 0.51)	0.09(0.07 ~ 0.12)	0.09(0.07 ~ 0.12)	3.33(1.83 ~ 5.24)	0.27(0.11 ~ 0.41)
3.2 ≤ DAS28 < 5.1	12.74(11.17 ~ 14.19)	1.35(0.96 ~ 1.79)	1.14(0.67 ~ 1.60)	0.41(0.25 ~ 0.61)	0.08(0.07 ~ 0.14)	0.10(0.08 ~ 0.14)	6.01(3.15 ~ 6.88)	0.42(0.21 ~ 0.70)
DAS28 ≥ 5.1	19.78(16.70 ~ 21.47)	1.72(1.25 ~ 2.24)	1.19(0.58 ~ 1.90)	0.42(0.27 ~ 0.57)	0.09(0.07 ~ 0.12)	0.12(0.10 ~ 0.15)	6.81(5.19 ~ 8.60)	0.57(0.21 ~ 0.70)
*p*	0.0001	0.0001	0.0570	0.3750	0.6496	0.0056	0.0001	0.0001

*RA* rheumatoid arthritis, *PFOA* Perfluorooctanoate, *PFNA* Perfluorononanoate, *PFDA* Perfluorodecanoate, *PFUnA* Perfluoroundecanoate, *PFDoA* perfluorododecanoate, *PFTrA* Perfluorotrdecanoate, *PFOS* Perfluorooctanesulfonate, *8:2Cl-PFESA* 8:2 Chlorinated polyfluorinated ether sulfonate, *DAS28* disease activity score in 28 joints based on c reactive protein, *M* mean, *SD* standard deviation

### Correlation between fluorides and BMI

Fluorides can infect the human beings Body Mass Index (BMI). We investigated the distribution of fluoride concentrations in different BIM groups. The patients were divided into three groups according to BMI, low weight group (BMI < 18.5 kg/m^2^), normal weight group (18.5 kg/m^2^ ≤ BMI < 24.9 kg/m^2^), and high weight group (BMI ≥ 25 kg/m^2^). The median serum concentration of PFOA (14.69 ng/mL, 7.63 ng/mL, 4.01 ng/mL), PFNA (1.70 ng/mL, 1.27 ng/mL, 1.13 ng/mL), and PFOS (5.98 ng/mL, 4.99 ng/mL, 2.78 ng/mL) reached statistical difference in low weight group, normal weight group, and high weight group with *p* = 0.0001, *p* = 0.0007 and *p* = 0.0005 respectively. Although it was different statistically (*p* = 0.0225), the median serum concentration of PFTrA, was 0.11 ng/mL, 0.10 ng/mL, and 0.14 ng/mL in low weight group, normal weight group, and high weight group respectively, did not correlated linearly with the BMI. The median serum concentration of PFDA, PFUnA and PFDoA did not reach the statistical difference in different BMI groups with *p* = 0.1149, *p* = 0.3380 and *p* = 0.5405 respectively. The detailed data was shown in the Table 3.

**Table 3:** Correlation between fluoride and BMI

	PFOA	PFNA	PFDA	PFUnA	PFDoA	PFTrA	PFOS	8:2Cl-PFESA
BMI < 18.5	14.69(12.63 ~ 19.37)	1.70(1.35 ~ 2.31)	0.45(0.50 ~ 1.63)	0.38(0.24 ~ 0.57)	0.09(0.07 ~ 0.14)	0.11(0.08 ~ 0.14)	5.98(3.37 ~ 7.16)	0.34(0.18 ~ 0.62)
18.5 ≤ BMI < 24	7.63(6.25 ~ 11.17)	1.27(0.97 ~ 2.03)	0.78(0.46 ~ 1.13)	0.36(0.20 ~ 0.45)	0.09(0.07 ~ 0.10)	0.10(0.07 ~ 0.14)	4.99(2.17 ~ 6.74)	0.29(0.13 ~ 0.58)
BMI ≥ 24	4.01(3.30 ~ 6.23)	1.13(0.64 ~ 1.52)	0.93(0.51 ~ 1.45)	0.41(0.25 ~ 0.54)	0.10(0.08 ~ 0.16)	0.14(0.10 ~ 0.16)	2.78(2.11 ~ 3.69)	0.18(0.11 ~ 0.52)
*p*	0.0001	0.0007	0.1149	0.3380	0.5405	0.0225	0.0005	0.1172

*BMI* body mass index, *PFOA*Perfluorooctanoate, *PFNA* Perfluorononanoate, *PFDA* Perfluorodecanoate, *PFUnA* Perfluoroundecanoate, *PFDoA* perfluorododecanoate, *PFTrA* Perfluorotrdecanoate, *PFOS* Perfluorooctanesulfonate, *8:2Cl-PFESA* 8:2 Chlorinated polyfluorinated ether sulfonate

### Correlation between fluorides and hematological damage

We investigated the distribution of fluoride concentrations in different White Blood Cell (WBC) groups and in different hemoglobin (Hb) groups. The patients were divided into two groups according to the count of WBC in blood, leukopenia group (WBC < 4 × 10^9^/L) and none leukopenia group (WBC ≥ 4 × 10^9^/L). The median serum concentration of PFOA (10.38 ng/mL, 6.19 ng/mL) and PFOS (5 ng/mL, 2.21 ng/mL) reached statistical difference in leukopenia group and none leukopenia group with *p* = 0.0215 and *p* = 0.0484 respectively. There was no statistically significant difference between the two groups for the remainder fluorides. The detailed data was shown in the Table 4.

**Table 4:** Correlation between fluorides and hematological damage

		PFOA	PFNA	PFDA	PFUnA	PFDoA	PFTrA	PFOS	8:2Cl-PFESA
WBC (× 10^9^/L)	≥ 4.0	10.38(5.26 ~ 15.29)	1.44(0.87 ~ 2.19)	0.91(0.33 ~ 1.45)	0.36(0.18 ~ 0.52)	0.09(0.07 ~ 0.12)	0.11(0.07 ~ 0.14)	5(2.17 ~ 6.82)	0.29(0.14 ~ 0.58)
	< 4.0	6.19(3.11 ~ 7.15)	1.19(0.7 ~ 1.67)	0.97(0.53 ~ 1.56)	0.35(0.24 ~ 0.68)	0.09(0.07 ~ 0.13)	0.13(0.07 ~ 0.16)	2.21(1.85 ~ 3.58)	0.19(0.12 ~ 0.57)
	*p*	0.0215	0.1390	0.7263	0.9422	0.2833	0.8013	0.0484	0.6113
Hb (g/L)	≥ 100	9.44(5.795 ~ 15.44)	1.48(1.03 ~ 2.14)	0.92(0.46 ~ 1.37)	0.34(0.20 ~ 0.50)	0.09(0.07 ~ 0.12)	0.11(0.08 ~ 0.14)	4.50(2.31 ~ 6.78)	0.36(0.14 ~ 0.57)
	< 100	10.87(6.21 ~ 14.94)	1.44(1.11 ~ 2.24)	1.03(0.51 ~ 1.41)	0.42(0.29 ~ 0.54)	0.09(0.07 ~ 0.16)	0.11(0.08 ~ 0.15)	5.19(2.79 ~ 6.67)	0.25(0.14 ~ 0.54)
	*p*	0.3887	0.4000	0.5663	0.0423	0.5297	0.2555	0.4732	0.5598

*PFOA* Perfluorooctanoate, *PFNA* Perfluorononanoate, *PFDA* Perfluorodecanoate, *PFUnA* Perfluoroundecanoate, *PFDoA* perfluorododecanoate, *PFTrA* Perfluorotrdecanoate, *PFOS* Perfluorooctanesulfonate, *8:2Cl-PFESA* 8:2 Chlorinated polyfluorinated ether sulfonate, *WBC* white blood cell, *Hb* hemoglobin

We also investigated the distribution of fluoride concentrations in different Hb groups. The patients were divided into two groups according to the concentrations of Hb in blood, hemoglobinopenia group (Hb < 100 g/L) and non hemoglobinopenia group (Hb ≥ 100 g/L). The median serum concentration of PFUnA was 0.42 ng/mL and 0.34 ng/mL in hemoglobinopenia group and non hemoglobinopenia group respectively with *p* = 0.0215 and *p* = 0.0484 respectively. There was no statistically significant difference between the two groups for the remainder fluorides. The detailed data was shown in the Table 4.

### Correlation between fluorides and ILD

Interstitial lung disease (ILD) is one of the most important visceral lesions in patients with RA. The patients were divided into two groups depending on with or without ILD. The study showed the median serum concentration of PFDA and PFUnA was 0.99, 0.51 and 0.38, 0.31 in the ILD group and none ILD group, and the difference was statistically significant with *p* = 0.0173 and *p* = 0.0254. There was no statistically significant difference between the two groups for the remainder fluorides. The detailed data was shown in the Table 5.

**Table 5:** Correlation between fluorides and ILD

		PFOA	PFNA	PFDA	PFUnA	PFDoA	PFTrA	PFOS	F-53B	8:2Cl-PFESA
ILD	+	9.79(5.03 ~ 15.19)	1.39(0.87 ~ 2.25)	0.99(0.38 ~ 1.58)	0.38(0.19 ~ 0.56)	0.09(0.07 ~ 0.12)	0.11(0.08 ~ 0.14)	4.26(2.12 ~ 6.68)	3.17(1.59 ~ 5.25)	0.29(0.14 ~ 0.57)
	–	8.94(6.05 ~ 17.44)	1.44(1.069 ~ 2.1)	0.51(0.34 ~ 0.96)	0.31(0.18 ~ 0.39)	0.09(0.06 ~ 0.11)	0.08(0.06 ~ 0.16)	5.19(1.876 ~ 6.81)	3.17(2.089 ~ 5.17)	0.28(0.12 ~ 0.6)
	*p*	0.7987	0.3099	0.0173	0.0254	0.9869	0.3403	0.4913	0.9929	0.9205

*ILD* interstitial lung disease*, PFOA*Perfluorooctanoate, *PFNA* Perfluorononanoate, *PFDA* Perfluorodecanoate, *PFUnA* Perfluoroundecanoate, *PFDoA* perfluorododecanoate, *PFTrA* Perfluorotrdecanoate, *PFOS* Perfluorooctanesulfonate, *8:2Cl-PFESA* 8:2 Chlorinated polyfluorinated ether sulfonate

## Discussion

It is considered that environmental factors acting on individuals with genetic susceptibility to cause immune disorders is the basic pathogenesis of initiation of RA. Studies have shown that smoking, alcohol consumption, microbial infection and contraceptives are important environmental factors in the onset of RA. Among them, smoking was the strongest environmental factor associated with RA, and the risk of RA in smokers was 2 to 4 times higher than in non-smokers [28]. The effect of smoking on RA showed a dose-dependent effect, and the longer smoking time, the greater the risk of RA [29]. The risk of RA decreases as the time to quit smoking increases, with abstinence for 15 years reducing the risk by 30 percent compared with abstinence for 1 year [30]. Smoking induces the production of anti-citrullinated Protein antibody (ACPA) in the lungs of RA patients who smoke but not non-smokers [31]. While, alcohol consumption may be a protective factor for RA, and those who consumed 500 g of white wine per week had a 50 percent lower risk of RA than those who consumed light or no alcohol [32]. Infection, especially viral infection, is also a major environmental factor for RA [33]. Pathogenic microorganisms can both trigger autoimmune responses through molecular mimicry mechanisms and induce citrullination processes, required for the production of ACPA. Recent studies have shown that oral flora, especially periodontal disease caused by *porphyromonas gingivalis*, is closely related to the pathogenesis of RA [34]. Several studies evaluated the role of particulate pollutants in the development of RA, indicating that some particulate pollutants can be treated as antigens by airway epithelial cells and presented to immune cells. Workers exposed to asbestos and silica have a higher risk of RA, suggesting that inorganic dust and CS may play specific roles in inducing disease [35].

Other environmental factors may also influence RA. Fluoride was widely used in production and life at some time ago, and it has been shown to have some effect on human health. Some studies had showed the potential immunomodulatory effects of exposure to Perfluoroalkyl substances (PFASs), and most of them were from studies about associations between exposure to PFASs and vaccine antibody response [36, 37]. Some other studies showed that some infectious and allergic diseases correlated with exposure to PFASs [38, 39]. Studies have suggested that perfluoroalkyl substances can affect the human immune system and associated with other autoimmune disease. A investigation in the Taiwanese GBCA reported positive associations between concurrent serum PFAS concentrations and levels of T helper 2 cytokines [IL-4 and IL-5] and negative associations with T helper 1 cytokines (interferon-γand IL-2) [40]. Another study showed that higher concentrations of multiple PFASs were significantly associated with lower odds of detectable IL-1β, and perfluoroalkyl carboxylic acids had inverse associations with TNF-α, whereas the perfluoroalkyl sulfonic acids showed positive associations[41]. Perfluoroalkyl substances can affect the expression of several immunomodulatory genes (CYTL1, IL27) as well as other immune-associated genes (e.g. EMR4P, SHC4, ADORA2A) [42]. So, it is natural to think of a possible association between fluoride and autoimmune diseases, such as RA.

In this study we initially looked at the effect of fluoride on patients with rheumatoid arthritis. We described the demographic, clinical, and Immunologic Features of 155 Chinese Patients With Rheumatoid Arthritis. Most of the patients were female, and the average age of onset was about 43. The average duration of the disease when entering the study was 12 years and most of them had moderate to high disease activity. In the visceral system damage, the most common was the involvement of the blood system, liver and kidney function damage was relatively rare. Interstitial lung disease was not uncommon (Table 1).

In this study, we detected 8 fluorides, PFOA, PFNA, PFDA, PFUnA, PFDoA, PFTrA, PFOS, 8:2Cl-PFESA, and these fluorides are higher in the serum of RA patients than in health controls. We found DAS28 related positively with the PFOA, PFNA, PFOS and 8:2Cl-PFESA. We analyzed the relationship between these fluorides and some common clinical indicators of RA. The median serum concentration of PFOA, PFNA, PFOS and 8:2Cl-PFESA in disease inactivity group, low disease activity group, moderate disease activity group and high disease activity group reached statistical difference with all *p* = 0.0001. But the median serum concentration of PFDA (*p* = 0.0570), PFUnA (*p* = 0.3750), and PFDoA (*p* = 0.6496) was not different statistically in different groups. Although it was different statistically (*p* = 0.0056), the median serum concentration of PFTrA did not correlated linearly with the increase of DAS28. The median serum concentration of PFDA, PFUnA and PFDoA did not reach the statistical difference in different DAS28 groups (Table 2). The data showed fluorides may promote the disease activity. Previous studies have shown that perfluoroalkyl substances can affect a human being's weight, white blood cell and lymphocyte counts, and hemoglobin [43]. Therefore, we investigated the effects of perfluoroalkyl substances on the above indexes in patients with RA, as well as the effects of a visceral involvement, interstitial pneumonia, which is common in rheumatoid arthritis. The median serum concentration of PFOA, PFNA, and PFOS reached statistical difference in low weight group, normal weight group, and high weight group with *p* = 0.0001, *p* = 0.0007 and *p* = 0.0005 respectively. Although it was different statistically (*p* = 0.0225), the median serum concentration of PFTrA did not correlated linearly with the BMI. The median serum concentration of PFDA, PFUnA and PFDoA did not reach the statistical difference in different BMI groups (Table 3). These data suggest that fluoride may affect nutrient metabolism in RA patients. We also investigated the effects of fluoride on the blood system. The median serum concentration of PFOA and PFOS reached statistical difference in leukopenia group and none leukopenia group with *p* = 0.0215 and *p* = 0.0484 respectively. The median serum concentration of PFUnA reached the statistical difference in hemoglobinopenia group and non hemoglobinopenia group (Table 4). Interstitial lung disease (ILD) is one of the most important visceral lesions in patients with RA. The study showed the median serum concentration of PFDA and PFUnA reached the statistical difference between the none ILD group and ILD group (Table 5). This suggests that fluoride not only affects the disease activity of RA, but may also promote the occurrence of complications, such as blood system abnormality and ILD.

In conclusion, our study showed that there was a close relationship between fluoride and RA, suggesting that fluoride may be an important environmental factor that promotes the onset and progression of RA. However, this study also has great limitations. This study only observed the correlation between fluoride and the clinical manifestations of RA, and further studies are needed to explore the possible mechanism of fluoride promoting the occurrence and development of RA.

## Conclusion

Some fluorides are closely related to the clinical manifestations of rheumatoid arthritis. We speculate that fluoride may be an important environmental factor contributing to the onset of RA and may lead to immune disorders in genetically predisposed individuals, leading to the onset or progression of rheumatoid arthritis.Further study should be made on the mechanism of fluoride leading to immune disorder and thus to the disease.

## Methods

### Patients and serum collection

We recruited 155 patients with RA and 145 health controls in the Zhejiang University School of Medicine Second Affiliated Hospital between March 2019 and Feb 2020. Patients were diagnosed as RA according to the EULAR/ACR 2010 criteria for RA and recruited during a routine rheumatology clinic assessment. There is no evidence that the participants had been occupationally exposure to PFASs. Interstitial lung disease (ILD) was indicated by the presence of fibrosis in computed tomography (CT) scans. Prior to blood sampling, each participant provided a signed informed consent at the time of registration. The current research agreement was approved by the Ethic Committee of the Zhejiang University School of Medicine Second Affiliated Hospital (ethics approval number: I2020001799).

Blood sample of participants were collected by physicians or nurses in BD-Vacutainer collection tubes (Becton, Dickinson and Company, NJ, USA) from cubital vein with Anticoagulant. After a brief mixed, the whole blood samples were centrifuged at 3000 r/min for 10 min to separate serum components and transferred to new blood tubes. The separated serum samples that stored in liquid nitrogen tank were then transported to the laboratory. Preparing field blanks (200 μL Milli-Q water, n = 3) were shipped with real serum samples during the sampling process. All collected serum specimen were stored at – 80 °C until analysis.

### Standards, reagents, and nomenclatures

All Certified standards of br-PFOSK, br-PFHxSK, MPFAC-MXA, PFAC-MXB, 8:2Cl-PFAES, 6:2Cl-PFAES and T-PFOA were obtained from Wellington Laboratories (Guelph, ON, Canada). br-PFOSK, br-PFHxSK and T-PFOA are derivatives of PFOS, PFHxS, PFOA, respectively, which provided by 3 M Co. and based on ^19^F-NMR analysis, the relative percentage of linear and branched isomers has precise data. PFAC-MXB consists of various PFAA standard solutions, including PFOA, perfluoropentanoate (PFPeA), perfluorododecanoate (PFDoA), perfluorobutanoate (PFHxA), perfluoroheptanoate (PFHpA), perfluorononanoate (PFNA), perfluoroundecanoate (PFUnA), perfluorobutanoate (PFBA), perfluorohexane sulfonate (PFHxS), perfluorotrdecanoate (PFTrA), perfluorotetradecanoate (PFTeA), perfluorobutane sulfonate (PFBS), perfluorodecanoate (PFDA), and PFOS. MPFAC-MXA is the mixture of eight isotopically-labeled standards (i.e., ^13^C_4_-PFOS, ^13^C_2_-PFHxA, ^13^C_5_-PFNA, ^13^C_2_-PFDA, ^13^C_2_-PFUnA, ^13^C_4_-PFOA, ^13^C_2_-PFDoA, ^18^O_2_-PFHxS, and ^13^C_2_-PFDoA).

Acetonitrile, Methanol (HPLC-grade), formic acid, and ammonium acetate were obtained from J&K Scientific Co. Ltd. (shanghai, China) and ANPEL Laboratory Technologies Inc. (Shanghai, China). Milli-Q water (18 MΩ) was used in the laboratory (Academic A10; Germany).

For PFAS isomers (i.e. PFOA, PFOS, and PFHxS isomers), we adopt the nomenclatures from our previous study [44]. Take PFOA as an example, we are abbreviated the linear, sum of all branched isomers, perfluoroisopropyl, 3-perfluoromethyl, 4-perfluoromethyl, and 5-perfluoromethyl as n-PFOA, br-PFOA, iso-PFOA, 3 m-PFOA, 4 m-PFOA, and 5 m-PFOA, respectively.

### Sample extraction

Serum samples were extracted following an acetonitrile-based extraction method [45], with slightly modifications. Prior to extraction, 200 μL of serum sample was spiked with internal standards (1.5 ng each), and then shook it by hands until completely mixed. After that, 4 mL acetonitrile were added to the serum samples. The mixture was vortexed, sonicated (53 kHz) for 30 min, and centrifuged at 4000 r/min for 10 min. The supernatant was transferred to a new 10 mL polypropylene (PP) tube (Biosharp, Beijing, China).The above extraction step was repeated once again with 4 mL of Methanol. The eluent was evaporated to near-dryness using gentle nitrogen. The residue was reconstituted in 200 μL of Methanol for HPLC-tandem mass spectrometry (MS/MS) analysis.

### Instrumental analysis

Serum samples were analyzed for 19 PFASs using high-performance liquid chromatography with tandem mass spectrometry (HPLC–MS/MS), as described in Grandjean et al. [46]. 10 μL of each sample extract was injected onto an ACQUITY UPLC BEH C_18_ column (1.7 μm, 2.1 mm × 50 mm; Waters, Miford, USA) at 40 °C. The mobile phase was constituted by 2 mM ammonium acetate (A) and methanol (B). The UPLC flow rate was 0.2 mL/min and the elution gradient started at 80% A and 20% B, then ramped up to 50% B by 12 min and was held at 100% B for 2 min; finally, it was returned to the initial condition. The mass spectrometer was operated in the electrospray negative ionization mode. Recording chromatograms through multiple reaction monitoring. The parent and product ions of target PFASs are listed in Additional file 1: Table S1 of the Supporting Information (Additional file 1: Table S2).

### Quality assurance and quality control

In order to monitor any method contamination, procedure blanks (i.e., 200 μL of Milli-Q water, n = 3) and quality control samples was identically performed for the pretreatment of actual human serum samples. When specific PFASs were not detected in the blank samples, limits of detection (LODs) was defined as the addition level corresponding to a signal-to-noise ratio of three. If the analytes that were detected in the blank samples, LODs was reported as the mean value of the concentration plus three times the standard deviation of the blank. Triplicate recovery experiments were performed with spiked samples including human and fetal bovine serum samples. The mean recoveries of main PFASs in human serum were satisfaction, which were in the range of 70–120% and the relative standard deviations (RSD) of recoveries were less than 10%.

### Statistical analysis

Descriptive data are presented as means ± standard deviation for continuous variables when the data were normally distributed or as M (P25–P75) when the data were non-normally distributed; numbers (%) are indicated for the categorical variables. Continuous variables were analyzed with Student t test in large samples of similar variance, or with the nonparametric Mann–Whitney U test for small samples. Categorical data were compared using the χ^2^ or Fisher exact tests. A 2-tailed value of P < 0.05 indicated statistical significance. Kruskal–Wallis test was used to compare the variables of multiple groups. Statistical analyses were performed with the 16.0 Stata/MP program (StataCorp LP, College Station, TX).

## Supplementary Information

**Additional file 1.** Supplementary materials.

## Data Availability

The datasets generated and analysed during the current study are not publicly available due to without the consent from all patients to publish this data, but are available from the corresponding author on reasonable request.

## Abbreviations

PFASs: Perfluoroalkyl substances
RA: Rheumatoid arthritis
PFOA: Perfluorooctanoate
PFNA: Perfluorononanoate
PFTrA: Perfluorotrdecanoate
PFOS: Perfluorooctanesulfonate
BMI: Body mass index
8:2Cl-PFESA: 8:2 Chlorinated polyfluorinated ether sulfonate
DAS28: Disease activity score 28
CRP: C reactive protein
PFUnA: Perfluoroundecanoate
PFDA: Perfluorodecanoate
HLA: Human leukocyte antigen
PTPN22: Protein tyrosine phosphatase non-receptor type 22
PADI4: Peptidylarginine deiminase 4
ACPA: Anti-citrullinated peptide antibodies
NHANES: National Health and Nutrition Examination Survey
EULAR: European League against Rheumatism
ACR: American College of Rheumatology
ILD: Interstitial lung disease
CT: Computed tomography
br-PFOSK: Br-Potassium perfluorooctane sulfonate
br-PFHxSK: Br-Potassium perfluorohexane sulfonate
PFPeA: Perfluoropentanoate
PFDoA: Perfluorododecanoate
PFHxA: Perfluorobutanoate
PFHpA: Perfluoroheptanoate
PFBA: Perfluorobutanoate
PFHxS: Perfluorohexane sulfonate
PFTeA: Perfluorotetradecanoate
PFBS: Perfluorobutane sulfonate
HPLC: High performance liquid chromatography
RSD: Relative standard deviations
