# Supplementary material for: The influences of perfluoroalkyl substances on the rheumatoid arthritis clinic
Source: BMC Immunol. 2022 Mar 4;23:10. doi: 10.1186/s12865-022-00483-7 (PMC8895528; doi:10.1186/s12865-022-00483-7)
Supplement: Supplementary file 1 — Additional file 1. Supplementary materials. [file 12865_2022_483_MOESM1_ESM.docx]

**[Supplementary](javascript:;) [tables](javascript:;)**

Table S1. Comparison of fluorinated compounds in RA patients and healthy controls (M(P20~P50) ng/ml)

| group | PFOA | PFNA | PFDA | PFUnA | PFDoA | PFTrA | PFOS | F-53B | 8:2Cl-PFESA |
| --- | --- | --- | --- | --- | --- | --- | --- | --- | --- |
| control | 3.94(2.64~5.39) | 1.19(0.77~1.57) | 0.93(0.36~1.45 | 0.36(0.18~0.54) | 0.09(0.07~0.12) | 0.11(0.07~0.14) | 2.2(1.34~3.04) | 1.17(0.68~1.9) | 0.08(0.06~0.11) |
| RA | 9.6(5.15~15.22) | 1.4(0.82~2.17) | 1.48(0.89~2.06) | 0.83(0.44~1.34) | 0.11(0.08~0.14) | 0.21(0.12~0.03) | 2.65(2.11~6.74) | 3.17(1.59~5.23) | 0.41±0.04 |
| *p* | 0.0000 | 0.0032 | 0.0000 | 0.0000 | 0.0008 | 0.0000 | 0.0000 | 0.0000 | 0.0000 |

| Nomenclature and acronym | Parent ion (*m/z*) | Product ion (*m/z*) | Allocation of internal standards |
| --- | --- | --- | --- |
| ***Native standards*** | | |  |
| Perfluorooctanoate (PFOA) | 413 | 369, 269, 219 | ^13^C_4_-PFOA |
| Perfluorononanoate (PFNA) | 463 | 419, 219 | ^13^C_5_-PFNA |
| Perfluorodecanoate (PFDA) | 513 | 469, 269 | ^13^C_2_-PFDA |
| Perfluoroundecanoate (PFUnA) | 563 | 519, 319 | ^13^C_2_-PFUnA |
| Perfluorododecanoate (PFDoA) | 613 | 569, 419 | ^13^C_2_-PFDoA |
| Perfluorotrdecanoate (PFTrA) | 663 | 619, 319 | ^13^C_2_-PFDoA |
| Perfluorooctane sulfonate (PFOS) | 499 | 80, 99, 130 | ^13^C_4_-PFOS |
| ***Internal Standards*** | | |  |
| Perfluoro-*n*-[1,2,3,4-^13^C_4_]-octanoic acid  (^13^C_4_-PFOA) | 417 | 372 |  |
| Perfluoro-*n*-[1,2,3,4,5-^13^C_5_]-nonanoic acid  (^13^C_5_-PFNA) | 468 | 423 |  |
| Perfluoro*-n*-[1,2-^13^C_2_]-decanoic acid  (^13^C_2_-PFDA) | 515 | 470 |  |
| Perfluoro-*n*-[1,2-^13^C_2_]-undecanoic acid  (^13^C_2_-PFUnA) | 565 | 520 |  |
| Perfluoro-*n*-[1,2-^13^C_2_]-dodecanoic acid  (^13^C_2_-PFDoA) | 615 | 570 |  |
| Perfluoro-1-octane-[1,2,3,4-^13^C_4_]- sulfonate  (^13^C_4_-PFOS) | 503 | 80, 99 |  |

Table S2. Perfluoroalkyl Substances Monitored in the Present Study and Their Acronyms, Parent Ions, and Product Ions.
